# The Binge Eating Scale: Structural Equation Competitive Models, Invariance Measurement Between Sexes, and Relationships With Food Addiction, Impulsivity, Binge Drinking, and Body Mass Index

**DOI:** 10.3389/fpsyg.2019.00530

**Published:** 2019-03-22

**Authors:** Tamara Escrivá-Martínez, Laura Galiana, Marta Rodríguez-Arias, Rosa M. Baños

**Affiliations:** ^1^Department of Personality, Evaluation and Psychological Treatment, Faculty of Psychology, University of Valencia, Valencia, Spain; ^2^Polibienestar Institute, University of Valencia, Valencia, Spain; ^3^Department of Methodology for the Behavioral Sciences, Faculty of Psychology, University of Valencia, Valencia, Spain; ^4^Department of Psychobiology, Faculty of Psychology, University of Valencia, Valencia, Spain; ^5^CIBER Fisiopatología Obesidad y Nutrición (CB06/03), Instituto Carlos III, Madrid, Spain

**Keywords:** binge eating, psychometric properties, confirmatory factor analysis, college students, sexes, convergent validity

## Abstract

**Introduction:** The Binge Eating Scale (BES) is a widely used self-report questionnaire to identify compulsive eaters. However, research on the dimensions and psychometric properties of the BES is limited.

**Objective:** The aim of this study was to examine the properties of the Spanish version of the BES.

**Methods:** Confirmatory Factor Analyses (CFAs) were carried out to verify the BES factor structure in a sample of Spanish college students (*N* = 428, 75.7% women; age range = 18–30). An invariance measurement routine was carried out across sexes, the latent means were compared, and estimates of reliability and convergent and discriminant validity were presented.

**Results:** A one-factor model fit the data best and was also equivalent between sexes. The scalar invariance model showed statistically significant differences across sexes, with a higher prevalence in women. Regarding reliability, the results were excellent. Finally, high statistically significant correlations were obtained with other measures of binge eating (BE), food addiction, impulsivity, binge drinking, and body mass index (BMI).

**Conclusion:** The Spanish 16-item BES is a valid and reliable scale to evaluate BE in the youth population.

## Introduction

Binge eating has become a serious problem worldwide with numerous consequences for general and clinical populations (Kessler et al., 2013). BE is characterized by the appearance of episodes of compulsive eating (BE) with two essential aspects, the ingestion of an excessive amount of food in a short time and a feeling of loss of control over eating. BE is also accompanied by emotional distress and the absence of compensatory behaviors (which are characteristic of bulimia nervosa) (American Psychiatric Association, 2013).

Binge eating is also common in the community population, and its prevalence has increased over time (de Freitas et al., 2008; Smink et al., 2014). In fact, international research reports very high BE prevalence rates in adolescents and young adults (Ribeiro et al., 2014; Goldschmidt et al., 2015), with higher prevalence rates in women than in men (Preti et al., 2009; Kessler et al., 2013). Croll et al. (2002) reported that 26% of young women and 13% of young men have experienced an episode of BE once in the past year. In turn, Smink et al. (2014) recently showed two peaks in the onset of BE, the first one immediately after puberty at an average age of 14, and the second one at the end of adolescence (19–24 years) (Stice et al., 2013).

Several studies offer evidence suggesting that the BE disorder is associated with psychiatric comorbidities. Thus, a large amount of research has established a well-defined relationship between BE and addiction disorders. In particular, recent research has found a positive correlation between BE (BES) and food addiction (YFAS) in both clinical (Imperatori et al., 2014) and non-clinical populations (Burrows et al., 2017). In addition, recent research also indicates the presence of comorbidity between BE and the use of alcohol and binge drinking in young people (Kessler et al., 2013; Laghi et al., 2014; Fouladi et al., 2015). Impulsivity has also been positively associated with BE (Steward et al., 2017; Mason et al., 2018) and could play an important role in the comorbidity between disordered eating behavior and excessive alcohol consumption in young students (Ocampo et al., 2012). Finally, a significant positive association between BE and BMI and obesity has consistently been observed. Evidence to date suggests that the relationship between BE and BMI and obesity occurs in both clinical and non-clinical populations (Hudson et al., 2007; Villarejo et al., 2012; Kessler et al., 2013; Duarte et al., 2015; Duncan et al., 2017; Mustelin et al., 2017). Psychiatric comorbidity with BE can increase its severity, chronicity, and resistance to any type of psychiatric treatment, and it has been associated with numerous medical conditions, a deterioration in quality of life, a greater risk of weight gain and obesity, and increased medical mortality (Ocampo et al., 2012; Kessler et al., 2013; Thornton et al., 2017).

The high prevalence of BE and the problems it causes have led to a need to establish instruments for its measurement. In this regard, several self-report questionnaires have been developed. Gormally et al. (1982) developed the BES, Yanovski (1993) developed the Questionnaire on Eating and Weight Patterns-R (QEWP-R), and Fairburn and Beglin (1994) developed the Eating Disorder Examination Questionnaire (EDE-Q). These instruments have been widely used to assess BE.

The BES (Gormally et al., 1982) was designed as a measure of severity (vs. diagnosis) of BE, with the additional property of evaluating its affective, cognitive, and behavioral manifestations. Research on the BES scale stems from its outstanding role as a screening measure in clinical (Freitas et al., 2006) and non-clinical populations (Duarte et al., 2015) to evaluate BE severity and intervention outcomes (Telch et al., 2001; Katterman et al., 2014). Studies carried out in the past decade, mainly with obese patients, have shown that the BES is very sensitive and specific in distinguishing between compulsive and normal eaters (Freitas et al., 2006; Grupski et al., 2013). In addition, a large number of investigations have confirmed that the BES shows good validity in both general (Meno et al., 2008; Gordon et al., 2012; Duarte et al., 2015) and clinical populations (Zúñiga and Robles, 2006; Dezhkam et al., 2009; Hood et al., 2013).

Despite the relevance of the BES in eating-disorder research, its factor structure is still controversial. Gormally et al. (1982) originally proposed a two-factor structure, dividing the items into cognitive and behavioral BE. Since then, the scale has been validated in French, Portuguese, English, Italian, Malay, and Spanish (Mexican) (Gormally et al., 1982; Ricca et al., 2000; Freitas et al., 2006; Zúñiga and Robles, 2006; Robert et al., 2013; Duarte et al., 2015; Brunault et al., 2016). Of these studies, only four have studied the factorial structure of the BES, with differing results. In the Mexican study, the only one carried out in a Spanish-speaking sample, the authors found a two-factor structure through exploratory means (Zúñiga and Robles, 2006). This structure, however, presented some problems because several items showed important cross-loadings and/or higher loadings in a different factor from the expected one. Similar results for the internal structure were presented in the Malay version (Robert et al., 2013). The authors tested the BES factorial structure with an exploratory factor analysis and posited a two-factor structure as the best solution for the data. Major problems of this study included retaining a factor that explained only 8.15% of the variance and, again, using varimax rotation. However, when the authors studied the sensitivity, specificity, and reliability of the BES, they found a unidimensional structure. More recently, Duarte et al. (2015) and Brunault et al. (2016) provided evidence of a one-factor structure in a sample of Portuguese women from the general population and in French non-clinical and clinical populations, respectively. Using CFA, Duarte et al. (2015) found a good fit for a one-factor structure of the BES, with appropriate reliability estimates for both the scale and the items, and good convergent validity. The Portuguese version of the BES was found to have high test–retest reliability. Brunault et al. (2016) also provided evidence of a one-factor structure by means of exploratory factor analysis. Again, reliability estimates were adequate. Previous studies carried out by Ricca et al. (2000) and Freitas et al. (2006), although not focused on the factorial structure, studied the sensitivity and specificity of the BES in Italian and Portuguese samples, respectively, with appropriate results when used as a unidimensional diagnostic instrument. Other recent studies focused on the BES factorial structure are presented by Imperatori et al. (2016) and Marek et al. (2016). The study by Marek et al. (2016) reported a good fit for a bifactorial solution, based on previous results found by Hood et al. (2013). However, these authors found a lack of incremental validity for the behavioral manifestation factor, and modest evidence for the feelings/cognitions factor. Therefore, Marek et al. (2016) defended the use of the BES as a unidimensional measure of BE severity. Imperatori et al. (2016), in the same direction, tested both the one-factor and competing two-factor models and found a comparable fit to the data. Therefore, they defended the one-factor model as the most parsimonious one.

The BES questionnaire has been widely used, but research on the factors and properties of the BES in the general population is still quite limited. In particular, no study has examined the psychometric properties of the BES in general populations of young men and women, and specifically, in the Spanish general population. In addition, results on its dimensionality are contradictory, and most research has examined its validity in specific samples, such as clinical samples or samples of women, especially obese women who undergo bariatric surgery to lose weight (Hood et al., 2013; Marek et al., 2016), or obese and overweight patients seeking weight loss treatment (Imperatori et al., 2016). There is a need for more evidence about the factorial structure of the scale and additional psychometric properties in non-clinical samples.

### Aims

The aim of this study is to examine the psychometric properties of the BES in a large sample of Spanish university students, following several steps: (1) to study the factor structure by means of competitive structural equation models, specifically CFA; (2) to test the measurement invariance of the BES between sexes; (3) to offer evidence of its reliability; and (4) to investigate its convergent and discriminant validity by describing its relationships with variables that have been associated with the BES, such as BE behavior, food addiction, impulsivity, use of alcohol/binge drinking, and BMI, as found in other studies (Fouladi et al., 2015; Burrows et al., 2017; Mustelin et al., 2017; Mason et al., 2018).

## Materials and Methods

### Sample

The sample consisted of 428 Spanish university students who voluntarily took part in the present study. The sample comprised female university students (*n* = 324, 75.7%), with an average age of 21.04 years (*SD* = 4.22), and male university students (*n* = 104; 24.3%), with an average age of 22.27 years (*SD* = 5.39). Thirty-four participants (7.9%) were underweight (BMI < 18.5), 330 (77.1%) had normal weight (18.5 ≥ BMI ≤ 24.99), 55 (12.9%) were overweight (25 ≥ BMI ≤ 29.99), and 11 (2.1%) were obese (BMI ≥ 30), according to the World Health Organization [WHO] (2000). Two participants did not respond. Female students had a mean BMI of 21.91 (*SD* = 2.98), and male students had a mean BMI of 23.46 (*SD* = 2.84). More information can be found in Table 1.

**Table 1:** Sociodemographic and clinical data.

	Female		Male	
	*N*	%	*N*	%
Sex	324	75.7	104	24.3

	***M***	***SD***	***M***	***SD***

Age	21.04	4.22	22.27	5.39
Weight (in kg)	58.57	8.91	73.76	9.28
Height (in cm)	1.63	0.06	1.77	0.07
BES	8.35	6.57	22.27	5.39
BE disorder	0.65	0.89	0.78	1.00
Food addiction	5.81	4.56	5.30	4.94
Motor impulsiveness	4.46	4.01	5.37	4.40
Non-planning	7.78	4.73	9.16	5.06
Attention	7.28	4.37	8.44	4.46
Binge drinking	0.46	0.69	0.52	0.66
BMI	21.91	2.98	23.46	2.84


*M, mean; SD, standard deviation; BES, Binge Eating Scale score; BE disorder, binge eating disorder as surveyed in EDI-3: “Have you ever engaged in binge eating (eaten a lot of food and felt like you couldn’t stop eating)?”; BMI, body mass index.*

### Measures

#### The Binge Eating Scale (BES; Gormally et al., 1982)

The BES is a self-administered questionnaire composed of 16 items: eight items that describe behavioral manifestations (for example, eating fast or consuming large amounts of food) and eight items on associated feelings and cognitions (for example, fear of not stopping eating). Each item has a response range from 0 to 3 points (0 = no severity of the BES symptoms, 3 = serious problems on the BES symptoms). Marcus et al. (1988) created a range of scores for the BES from 0 to 46 points: a score of less than 17 points indicates minimal BE problems; a score between 18 and 26 points indicates moderate BE problems, and a score of more than 27 points indicates severe BE problems. Psychometric properties of the BES in the Spanish population are considered in this study.

In this investigation, the Mexican version of the BES validated in the Spanish language was used (Zúñiga and Robles, 2006). This version of the scale was subjected to a rigorous cultural adaptation procedure. First, a Spanish–English bilingual translator who was not familiar with the questionnaire reviewed the translation. Second, a native Spanish speaker who knew the purpose of the study reviewed the translated BES elements. Later, we evaluated whether the scale items were understood properly by administering the BES to forty Spanish university students. The objective was to confirm that it was a simple scale for the general young population to understand. The Spanish version of the BES was an exact translation of the original English version; therefore, the decision was made to use the same scale. The final version of the BES and its instructions are contained in the Annex.

#### The Eating Disorder Inventory-3 (EDI-3; Clausen et al., 2011)

The EDI-3 consists of a brief self-report questionnaire designed to evaluate the risk variables and other variables associated with eating behavior disorders. It consists of 91 items grouped in 12 subscales. Participants completed all 26 items on the scale (drive for thinness, bulimia, body dissatisfaction, and BE disorder). In the present study, only item 26, measuring BE behavior, was used in this validation: “*Have you ever engaged in binge eating (eaten a lot of food and felt like you couldn’t stop eating)?*” The participants responded to the items on a 6-point Likert scale (0 = never; 6 = always). In this study, the validated Spanish version of the EDI-3 was used (Elosua and López-Jáuregui, 2012). The alphas for the complete scale in this sample were 0.903 for drive for thinness, 0.813 for bulimia, and 0.747 for body dissatisfaction.

#### The Modified Yale Food Addiction Scale (mYFAS; Flint et al., 2014)

The mYFAS (short version of the YFAS) is a brief, self-administered instrument designed to assess the signs of addictive eating behavior. It is composed of nine items, seven that evaluate the diagnostic criteria for food addiction, and two that assess clinically significant deterioration and distress. The questionnaire uses a Likert rating scale (0 = never; 4 = four or more times or daily). The YFAS had good psychometric properties in the general population (Gearhardt et al., 2009; Pedram et al., 2013). In this study, the nine items from the validated Spanish version of the YFAS (Granero et al., 2014) were extracted. The internal consistency coefficient found for the Spanish version of the mYFAS in this study was α = 0.769.

#### Barratt Impulsiveness Scale-15S (BIS-15S; Spinella, 2007)

The BIS-15 is a brief, self-administered scale consisting of 15 items subdivided in three dimensions (motor, non-planning, and attention) that evaluate impulsivity. Items are scored on a 4-point Likert scale (0 = rarely, 4 = always or almost always). In the present study, the validated Spanish version (BIS-15S) was used (Orozco-Cabal et al., 2010), which contains good psychometric qualities in terms of internal consistency, temporal stability, and internal structure. Estimates of internal consistency in the present study were 0.783 for motor impulsivity, 0.756 for non-planned impulsivity, and 0.701 for attentional impulsivity.

#### Binge Drinking

We used the most internationally supported measure to assess binge drinking. The participants answered the following question: “*Taking into account all types of alcoholic beverages, did you consume five or more drinks in a row (four if you are a woman) on at least one occasion in the past month? How many times in the past month?*” The participants responded on a 4-point Likert scale (0 = never; 4 = four or more times a week) (Kuntsche et al., 2006; Paul et al., 2011).

#### Body Mass Index (BMI)

The BMI was calculated by dividing the weight in kilograms by the square of the height in meters (BMI = weight [kg]/height [m^2^]) (World Health Organization [WHO], 2000).

#### Additional Information

Participants were required to provide data about their sex, age, education level, country of residence, weight (in kg), and height (in cm), along with filling out the other questionnaires.

### Procedure

Most of the participants were recruited in the classrooms of the University of Valencia, and some of them by email and social networks. The participants were informed about the study design, the voluntary nature of their participation, and the confidentiality of the data obtained. Thus, they were informed that the questionnaires would be answered online through a computer or mobile phone. Once they had received this information, the participants delivered their informed consent on paper and, subsequently, were provided with a link to access the online survey. Participants were asked to answer the online survey. The survey was conducted using the Lime Survey web platform ^1^, where the participants provided demographic data and answered the measures mentioned in the previous section. Later, a blind evaluator analyzed the self-reported responses. The data collection followed the ethical standards. The study was approved by the Ethics Committee of the University of Valencia and was carried out in accordance with the ethical standards of the 1964 Declaration of Helsinki (Procedure number: H1513854038939).

### Statistical Analyses

The first step consisted of the study of factorial validity using CFA. The two structures found in the literature were tested: a one-factor model (BE) and a model with two correlated factors (behavioral and emotional/cognitive BE). Model fit was assessed using the chi square statistic, the CFI, with values of more than 0.90 (ideally 0.95) indicating good fit, and the RMSEA, with values of 0.08 or less for an excellent fit (Hu and Bentler, 1999). For model fit comparison, CFI differences were estimated. Whereas Little (1997) argued that CFI differences of 0.05 could be considered negligible, Cheung and Rensvold (2002) recommended a more restrictive cut-off point of 0.01. Both contributions were taken into account in the current research.

Once the best fitting model had been retained, the structure was tested separately in samples of women and men. Because the model fitted both sets of data adequately, the invariance routine was developed. Measurement invariance of factor loadings, intercepts, and means were tested, as recommended by Thompson and Green (2006) and van de Schoot et al. (2012). First, we tested the configural model, in which a model with a one-factor structure was estimated in the two samples, women and men. This model is also called the baseline model because its fit is used as the baseline fit with which the other models are compared. Second, we evaluated weak or metric invariance. In this model, factor loadings are constrained across samples; that is, they become the same for men and women. When metric invariance holds, it means that both women and men attribute the same meaning to BE. Third, we tested strong or scalar invariance. In the scalar invariance model, the intercepts are constrained across samples. If the model is tenable, it means that the meaning of BE (the factor loadings) and the intercepts are equal across groups. Finally, because scalar invariance was found, we constrained latent means across samples. If this last model fits, it means that levels of BE are equal across sexes. The models were compared using both chi-square differences and CFI differences.

Due to the ordinal nature of the BES items, we employed WLSMV as the estimation method, as recommended by Muthén and Muthén (1998), Flora and Curran (2004), and Brown (2006).

Evidence of reliability of the proposed structure was also gathered: Cronbach’s alpha, McDonald’s omega, and the items’ homogeneity were estimated for both samples.

Finally, evidence of external validity was gathered by calculating the Pearson correlation between the total score on the BES and other related variables, such as an indicator of BE obtained from the EDI-3 (specifically, item number 26, “*Have you binge eaten (eaten a lot of food and felt like you couldn’t stop eating?*”), food addiction, impulsivity, binge drinking, and BMI.

Analyses were performed using Mplus version 8 (Muthén and Muthén, 1998) and SPSS version 24 software. Missing data were dealt with using FIML, which is the most recommended method for structural equation modeling (Finney and DiStefano, 2013).

## Results

**FIGURE 1:**
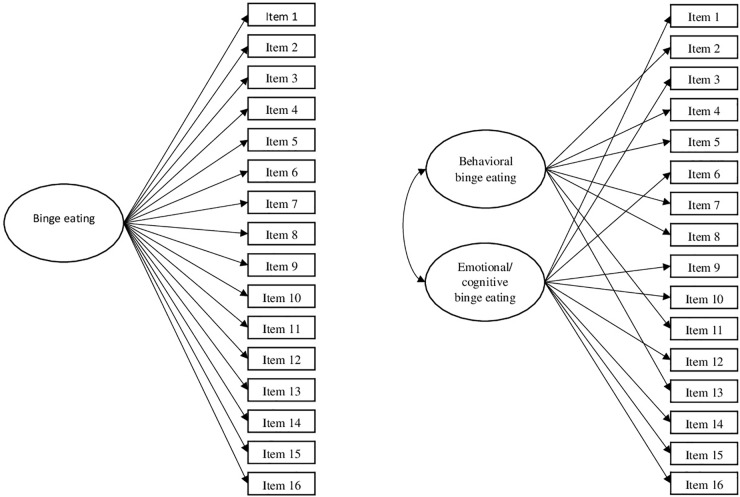
Confirmatory factor analyses (CFAs) models specified and tested for the Binge Eating Scale (BES) validation in the total sample.

Two competitive CFA, with the structures shown in Figure 1, were specified, estimated, and tested in the total sample. Table 2 shows the fit indexes for these models. Fit was excellent for both models (see Table 2). However, the correlation found between the two factors in the two-factor model was extremely high (*r* = 0.925 [0.912, 0.938]), and thus showed no discriminant validity. The CFI difference was not large (ΔCFI = 0.004); it was smaller than the one recommended by Little (1997) and the more restrictive one proposed by Cheung and Rensvold (2002). Taking all of this information into account, the one-factor solution, the most parsimonious one, was retained as the best representation of the data.

**Table 2:** Confirmatory factor analyses and set of nested models to test for measurement invariance.

	χ^2^	df	*p*	CFI	RMSEA	RMSEA CI	ΔCFI	Δχ^2^	Δdf	*p*
One-factor model in the total sample	374.349	104	<0.001	0.939	0.076	[0.067, 0.084]	–	–	–	–
Two-factor model in the total sample	359.458	103	<0.001	0.943	0.074	[0.066, 0.082]	0.004	–	–	–
One-factor model in women’s sample	321.494	104	<0.001	0.935	0.080	[0.070, 0.090]	–	–	–	–
One-factor model in men’s sample	134.841	104	0.022	0.975	0.053	[0.021, 0.077]	–	–	–	–
Configural invariance	430.278	208	<0.001	0.947	0.071	[0.061, 0.080]	–	–	–	–
Metric invariance	433.573	223	<0.001	0.950	0.066	[0.057, 0.076]	0.003	35.993	15	0.001
Scalar invariance	485.085	270	<0.001	0.949	0.061	[0.052, 0.070]	0.001	76.228	47	0.004
Scalar invariance with constrained latent means	547.693	271	<0.001	0.934	0.069	[0.061, 0.077]	–0.015	16.186	1	<0.001


*CFI, comparative fit index; RMSEA, root mean squared error of approximation; RMSEA CI, RMSEA 90% confidence interval.*

Once the one-factor model had been retained, the structure was tested separately in samples of women and men. The CFA of the BES tested in the samples of women and men separately showed an adequate fit (see Table 2).

Regarding the invariance routine results, the configural model fitted the data adequately (see Table 2). Thus, it was retained as the baseline model. When metric invariance was tested, statistically significant differences were found between the chi-squares, but the CFI and the RMSEA improved. Taking into account that in large samples the power of the chi square statistic to detect minor deviations is high (Fan et al., 1999), and the rest of the indices improved when factor loadings were constrained in the men’s sample, the metric invariance model was retained. Then, scalar invariance was tested, and again statistically significant differences were found between the chi-squares, but with a trivial decrease in the CFI (0.001) and an improvement in the RMSEA. Once again, the evidence guaranteed scalar invariance. Given that the BES has been found to be metric invariant, mean comparisons are meaningful, and they can be made at the latent level. When men’s mean on the BE factor was constrained to women’s, significant differences between the chi-squares were found, along with a significant decrease in the CFI (0.015) and an increase in the RMSEA. Thus, the scalar invariance model was retained as the most parsimonious model, and its factor loadings and intercepts are presented in Table 3. Regarding the level of BE, women showed higher levels: mean difference = 0.548, standard error = 0.144, *p* < 0.001, Cohen’s *d* = 0.548.

**Table 3:** Unstandardized and standardized factor loadings and intercept thresholds.

BES items	Factor loadings			Intercepts		
	UN	ST women sample	ST women sample	𝒱_ 1_	𝒱_2_	𝒱_3_
1	0.638	0.538	0.615	–0.386	0.944	2.755
2	0.567	0.492	0.570	0.020	0.514	2.094
3	1.162	0.758	0.818	0.576	1.929	3.732
4	0.680	0.562	0.640	–1.086	0.776	2.145
5	0.936	0.683	0.753	–0.253	1.823	3.326
6	0.845	0.646	0.719	0.187	1.921	3.318
7	1.240	0.778	0.835	1.409	2.533	3.551
8	0.863	0.653	0.726	–0.711	1.148	2.961
9	0.795	0.622	0.697	0.508	1.472	2.225
10	1.525	0.836	0.881	0.508	2.627	4.100
11	1.124	0.747	0.809	–0.048	2.431	3.834
12	0.646	0.543	0.620	0.802	1.748	2.635
13	0.380	0.355	0.422	0.148	1.391	1.729
14	1.086	0.736	0.799	–0.153	1.393	3.024
15	0.746	0.598	0.674	–0.285	1.099	2.141
16	0.637	0.537	0.615	0.665	1.835	2.992


*UN, factor loading unstandardized estimates (constrained to equality across samples); ST, factor loading standardized estimates; 𝒱, intercept threshold.*

Evidence of the reliability and internal consistency of the Spanish version of the BES was provided at scale and item levels. Cronbach’s alpha was 0.869, and McDonalds’ omega was 0.915, indicating appropriate reliability estimates for the scale. Descriptive statistics, item homogeneity, alpha if item-deleted, and inter-item correlations for the unidimensional model in the total sample are presented in Table 4.

**Table 4:** Means, standard deviations, item-adjusted total correlations, and alpha if item deleted for the one-factor model of the BES.

BES items	Total sample		Women sample				Men sample				r_it_	α_id_
	*M*	*SD*	*M*	*SD*	Skewness	Kurtosis	*M*	*SD*	Skewness	Kurtosis		
1	1.82	0.78	1.88	0.77	0.31	–0.92	1.53	0.68	1.07	0.71	0.470	0.863
2	1.80	0.94	1.81	0.94	0.51	–1.36	1.78	0.95	0.85	–0.48	0.389	0.870
3	1.43	0.68	1.45	0.69	1.33	0.78	1.35	0.63	1.82	3.00	0.625	0.856
4	2.07	0.74	2.14	0.72	0.42	0.23	1.81	0.77	0.58	–0.30	0.482	0.863
5	1.63	0.64	1.69	0.65	0.55	–0.07	1.43	0.61	1.38	2.03	0.570	0.859
6	1.48	0.63	1.55	0.63	0.80	–0.05	1.25	0.55	2.41	6.48	0.493	0.862
7	1.23	0.57	1.24	0.56	2.51	6.37	1.20	0.59	3.24	10.43	0.551	0.860
8	1.86	0.72	1.87	0.71	0.39	–0.30	1.81	0.74	0.45	–0.60	0.565	0.859
9	1.46	0.78	1.48	0.80	1.71	2.22	1.45	0.76	1.57	1.56	0.487	0.863
10	1.42	0.64	1.47	0.67	1.34	1.41	1.32	0.56	1.87	4.29	0.661	0.855
11	1.53	0.60	1.55	0.59	0.66	–0.03	1.47	0.62	1.21	1.62	0.612	0.858
12	1.31	0.62	1.33	0.62	1.93	3.38	1.25	0.63	2.75	7.35	0.445	0.864
13	1.55	0.77	1.58	0.78	1.45	1.85	1.50	0.80	1.82	3.09	0.309	0.871
14	1.69	0.78	1.76	0.78	0.71	–0.20	1.41	0.71	1.58	1.48	0.614	0.856
15	1.75	0.82	1.84	0.82	0.73	–0.09	1.55	0.79	1.44	1.57	0.511	0.862
16	1.33	0.59	1.35	0.59	1.55	1.78	1.25	0.58	2.53	6.31	0.432	0.865


*M, mean; SD, standard deviation; r_*it*_, item-adjusted total correlation; α_*id*_, alpha if item deleted.*

Finally, the results pointed to adequate convergent validity of the scale in the current sample, with a positive, high, and statistically significant correlation between the BES and the BE indicator from the EDI-3 (*r* = 0.621, *p* < 0.001). Regarding other related variables, relations were also positive and statistically significant with the dimensions of impulsivity: motor (*r* = 0.202, *p* < 0.001), non-planned (*r* = 0.164, *p* = 0.001), and attentional (*r* = 0.284, *p* < 0.001); food addiction (*r* = 0.761, *p* < 0.001); the binge-drinking indicator (*r* = 0.139, *p* = 0.023); and BMI (*r* = 0.243, *p* < 0.001).

## Discussion

Binge eating is a problematic clinical condition in young people. Studies have shown that youth is a critical stage for the onset of eating disorders, with the highest prevalence of the BE disorder at the beginning of young adulthood. The BES scale is one of the most widely used measures for screening and evaluating BE in both clinical and non-clinical samples. However, the factorial structure of the BES in the general population of men and women has not yet been evaluated.

The present study aimed to examine the psychometric properties of the Spanish version of the BES in young populations of men and women using several approaches. First, evidence of its factorial structure was gathered, following a competitive models approach. Second, and once the BES internal structure had been established, its measurement invariance was tested across sexes. Third, estimates of reliability were calculated. Finally, evidence of both convergent and discriminant validity was provided. These steps will guide the discussion.

Regarding evidence of the factorial structure, previous scientific evidence was taken into account, and both the one- and two-dimensional structures of the BES were tested. Our results supported the unidimensionality of the scale, with the best fitting model being the simplest structure. This result is consistent with recent results by Duarte et al. (2015) and Brunault et al. (2016), who also found a one-factor structure in the Portuguese and French versions of the scale. However, the previous study in the Spanish language had pointed to a structure with two-correlated factors (Zúñiga and Robles, 2006), also found in the Malay version (Robert et al., 2013). Although this was the original authors’ approach (Gormally et al., 1982), when tested here the results showed a high correlation between behavioral and cognitive BE. Thus, taking into account the trivial differences found in the models’ fit, we defend the one-dimensional structure.

Once the factor structure had been established, an invariance measurement routine was carried out in order to test whether the scale was invariant across sexes. The best fitting and more parsimonious model was the scalar invariant model. Thus, our results provide evidence of the absence of measurement bias when groups of women and men are compared. In the context of eating disorders, where women and men are usually viewed as different populations, measurement invariance becomes a core issue in making group comparisons (Kline, 2015). However, this condition is assumed, rather than tested, in most of the research carried out with the BES. In the original work, for instance, Gormally et al. (1982) tested mean differences across two samples (one with only females, and the other with females and males), but without offering evidence of BES invariance. In the same direction, Ricca et al. (2000) compared men’s and women’s scores on the BES, but with no previous test of measurement invariance, that is, with no guarantee of the absence of measurement bias.

The next step in the research, because scalar invariance held, was to compare latent means, and this was done by testing an additional model in which latent means were constrained. The model fit significantly decreased, and, thus, the results pointed to the existence of differences between means. Indeed, the scalar invariance model showed statistically significant differences between women and men, with a medium-sized difference, favoring the group of women. This result agrees with previous research that has revealed higher prevalence and mean scores on binge behavior in women (Ricca et al., 2000; Preti et al., 2009; Kessler et al., 2013). However, this is the first time this model has been tested in a latent mean context, that is, in an error-free measurement context.

Finally, estimates of reliability and convergent and discriminant validity are also provided. Regarding reliability, the results were excellent. In the case of convergent and discriminant validity, our evidence matched previous findings perfectly, with high and statistically significant correlations between the BES scores and the BE indicator from the EDI-3 and food addiction, and statistically significant but lower correlation values between the BES and the dimensions of impulsivity, binge drinking, and BMI (Freitas et al., 2006; Villarejo et al., 2012; Duncan et al., 2017; Steward et al., 2017; Mason et al., 2018). Our findings are also consistent with those presented by Imperatori et al. (2014) and Burrows et al. (2017) suggesting a strong association between BE and food addiction, and they corroborate previous evidence on the association between BE and alcohol/binge drinking (Kessler et al., 2013; Laghi et al., 2014; Fouladi et al., 2015). These results show the important clinical implications of understanding the relationship between BE and addictive behaviors, such as knowing what mechanisms underlie the appearance and development of these behaviors.

In summary, our findings indicate that the BES shows adequate psychometric properties when used in samples of Spanish females and males from a youth population. Indeed, this is the first time two competitive models have been tested for the internal structure of the BES, with evidence suggesting a one-dimensional structure, consistent with DSM-5 criteria (BE is defined by the rapid ingestion of an excessive amount of food and the loss of control over that ingestion, with discomfort with regard to BE and the absence of compensatory behaviors; American Psychiatric Association, 2013). As far as we know, this is the first study to evaluate the psychometric properties of the BES in the general youth population, including both males and females. To date, the only study that analyzed the BES factorial structure and psychometric properties in the general population was conducted by Duarte et al. (2015), but it only included a sample of Portuguese women. In addition, our study is the first one to test the scale’s measurement invariance across samples, in this case, across sexes.

These findings demonstrate that the Spanish version of the BES is a valid and reliable scale for the assessment of BE in a youth sample. This brief, easy-to-administer, self-report questionnaire consists of 16 items on one scale. It provides relevant information about clinically significant symptoms of BE, and it may be especially useful in prevention programs and community interventions for disordered eating behaviors.

This study contributes to a relevant line of research in the field of eating disorder evaluation and, specifically, BE. The present study confirms the unifactorial structure of the BES in a young community population of men and women. The BES is one of the most widely used scales worldwide in the detection of BE in both clinical and non-clinical samples. Its validation in a young general sample can help us to detect cases of BE in order to prevent a possible eating disorder and its associated medical comorbidities. In addition, this study provides data about the relationship between BE and other comorbid variables, helping us to better understand this prevalent problem in today’s society and, especially, in young people.

### Limitations and Future Research

Several limitations should be considered. First, only self-applied measures were used, and the participants may have suffered from social desirability bias. Thus, future research should include a semi-structured interview to obtain better reliability and specificity. Second, self-reported height and weight were used to calculate BMI. Several studies suggest that self-reported measures of weight and height should be viewed with caution because middle-aged men and women are more likely to exhibit biases; in particular, weight tends to be underestimated and height overestimated (Niedhammer et al., 2000), and these biases lead to underestimation of the BMI value. Third, it would have been interesting to test both versions of the questionnaire (English/Spanish) in bilingual students to obtain a stronger validation of the questionnaire. Fourth, all the participants were recruited from the University; therefore, the findings cannot be generalized to clinical settings. Several studies support the BES as a valid screening measure of BE in a non-clinical population (Duarte et al., 2015). In addition, there is a high prevalence of BE in the young general population, and so it was considered important to know the psychometric properties of the instrument in young people, in order to have a validated screening measure of BE in this population. However, future lines of research could perform invariance routines in clinical and non-clinical samples to verify whether the Spanish version of the BES has similar reliable results in clinical and non-clinical populations. Fifth, although the samples of women and men were unbalanced, the total size of the men’s sample did not allow us to adjust group size. This limitation will be taken into account in subsequent studies. In addition, although this study has shown that women have higher levels of BE than men, future studies should replicate this finding with a larger sample of men, in order increase the accuracy of the groups’ means. Sixth, due to problems of sample availability, the test–retest reliability could not be verified. Future studies should take this limitation into account. Finally, with regard to the one-dimensionality of the scale, future studies should explore whether the single dimension of the scale obtained in this study varies in other types of samples, for example, samples of older men and women, given the high prevalence of BE in this population (Hudson et al., 2007).

## Data Availability

The datasets generated for this study are available on request to the corresponding author.

## Author Contributions

TE-M contributed to preparing the measures and the protocol, researched the literature, recruited the participants and collected the data, and wrote the manuscript. LG contributed to the literature research, analyzed the data, and wrote the manuscript. MR-A and RB contributed to the design of the study and wrote the manuscript. All the authors contributed to manuscript revision and read and approved the submitted version.

## Conflict of Interest Statement

The authors declare that the research was conducted in the absence of any commercial or financial relationships that could be construed as a potential conflict of interest.

## Footnotes

1 encuestas.uv.es

## Annex: Cuestionario De Trastorno Por AtracÓN

Instrucciones: A continuación, se encuentran 16 grupos de tres o cuatro oraciones. Lea con cuidado cada una de las oraciones de cada grupo y marque con una «X» (cruz) la que mejor describa como se siente con respecto a los problemas que ha tenido para controlar su forma de comer.

### GRUPO 1

1. No me siento preocupado(a) de mi peso o mi talla cuando estoy con otros.
2. Me siento preocupado(a) de cómo luzco para los demás, pero normalmente esto no me hace sentirme decepcionado(a) de mí mismo(a).
3. Me siento preocupado(a) acerca de mi apariencia y peso y esto me hace sentir decepcionado(a) de mí mismo(a).
4. Me siento muy preocupado(a) acerca de mi peso y frecuentemente siento una pena intensa y disgusto por mí mismo(a). Trato de evitar contactos sociales debido a mi preocupación por mi apariencia.

### GRUPO 2

1. No tengo ninguna dificultad para comer lentamente y de manera adecuada.
2. Aunque parece que «devoro» la comida no termino sintiéndome «lleno(a)» por haber comido mucho.
3. Algunas veces como muy rápido y después me siento incómodamente lleno(a).
4. Siempre o casi siempre me paso la comida sin masticarla y cuando esto pasa me siento incómodamente «lleno(a)» porque he comido demasiado.

### GRUPO 3

1. Me siento capaz de controlar mis ganas de comer cuando yo quiero.
2. Siento que he fallado en controlar mi alimentación más que una persona promedio.
3. Me siento incapaz de controlar mis ganas de comer.
4. Me siento desesperado(a) porque no soy capaz de controlar mi manera de comer.

### GRUPO 4

1. No acostumbro comer cuando estoy aburrido(a).
2. ALGUNAS VECES me pongo a comer cuando estoy aburrido(a), pero SIEMPRE O CASI SIEMPRE soy capaz de mantenerme ocupado(a) y dejar de pensar en la comida.
3. SIEMPRE O CASI SIEMPRE me pongo a comer cuando estoy aburrido(a), pero ALGUNAS VECES puedo hacer otra actividad para dejar de pensar en la comida.
4. SIEMPRE O CASI SIEMPRE me pongo a comer cuando estoy aburrido(a) y nada parece ayudarme a romper este hábito.

### GRUPO 5

1. GENERALMENTE como cuando me siento físicamente con hambre.
2. ALGUNAS VECES como algo impulsivamente a pesar de que no tengo hambre.
3. MUCHAS VECES como cosas que realmente no disfruto para satisfacer mi sensación de hambre a pesar saber que físicamente no necesito comer en ese momento.
4. A pesar de que físicamente no tengo hambre, tengo una sensación de hambre en mi boca que sólo es satisfecha comiendo cosas que me llenan la boca, como un sándwich. Cuando hago esto, algunas veces escupo la comida para no engordar.

### GRUPO 6

1. No siento culpa ni me odio después de comer de más.
2. A VECES siento culpa o me odio después de comer de más.
3. SIEMPRE O CASI SIEMPRE siento culpa o me odio después de comer de más.

### GRUPO 7

1. Cuando hago dieta y la rompo porque como de más, puedo volver a controlar mi forma de comer.
2. Cuando hago dieta y la rompo comiendo algo «prohibido», ALGUNAS VECES siento que «me equivoqué» y como aún más.
3. Cuando hago dieta y la rompo porque como de más, MUCHAS VECES siento que «cometí un error» y como aún más.
4. SIEMPRE O CASI SIEMPRE hago dieta y la rompo porque tengo un atracón. Parece que mi vida transcurriera entre «atracones» y «tener hambre».

### GRUPO 8

1. MUY POCAS VECES O NUNCA como tanto como para sentirme incómodo(a).
2. ALGUNAS VECES, aproximadamente 1 vez al mes, como tanto que al final termino sintiéndome muy «lleno(a)».
3. MUCHAS VECES durante el mes tengo periodos donde como grandes cantidades de comida, ya sea a la hora de la comida o entre comidas.
4. SIEMPRE O CASI SIEMPRE como tanta comida que me siento incomodo(a) al acabar de comer, y a veces hasta con un poco de náuseas.

### GRUPO 9

1. Mi ingesta de calorías no es muy baja ni muy alta.
2. ALGUNAS VECES después de comer demasiado, trato de disminuir mi ingesta de calorías casi a cero para compensar el exceso que había comido.
3. SIEMPRE O CASI SIEMPRE como demasiado durante la noche. Generalmente no tengo hambre durante el día pero como demasiado en la noche.
4. Durante mi vida adulta hay semanas en que tengo largos periodos donde prácticamente me mato de hambre y luego como de más. Parece ser que mi vida transcurre entre «atracones» y «hambre».

### GRUPO 10

1. Normalmente soy capaz de parar de comer cuando yo quiero. Sé cuándo: «suficiente es suficiente».
2. ALGUNAS VECES me da compulsión de comer y parece que no puedo controlar mi manera de comer.
3. MUCHAS VECES me da una urgencia por comer y pareciera que no la puedo controlar, pero otras veces si la puedo controlar.
4. SIEMPRE O CASI SIEMPRE me siento incapaz de controlar mi urgencia por comer. Tengo miedo de no poder parar de comer cuando yo quiera.

### GRUPO 11

1. No tengo ningún problema para parar de comer cuando me siento «lleno».
2. Puedo parar de comer cuando me siento lleno, pero A VECES como demasiado y me siento «muy lleno».
3. MUCHAS VECES tengo el problema para parar de comer y me siento incómodamente «lleno».
4. SIEMPRE O CASI SIEMPRE soy incapaz de parar de comer cuando yo quiero y algunas veces ha sido necesario inducirme el vómito, usar laxantes o diuréticos para aliviar mi sensación de estar «muy lleno».

### GRUPO 12

1. Como lo mismo cuando estoy con otros (familia, reunión social) que cuando estoy solo.
2. ALGUNAS VECES cuando estoy con otras personas no como tanto como quisiera porque me siento preocupado(a) acerca de mi forma de comer.
3. MUCHAS VECES como pequeñas cantidades de comida cuando hay gente a mí alrededor porque me siento avergonzado de mi forma de comer.
4. SIEMPRE O CASI SIEMPRE me siento muy avergonzado(a) por comer de más y elijo tiempos para comer de más cuando sé que nadie podría verme. Me siento como «un tragón de closet».

### GRUPO 13

1. Hago tres comidas al día y SÓLO ALGUNAS VECES como bocadillos entre comidas.
2. Hago tres comidas al día, pero MUCHAS VECES como bocadillos entre éstas.
3. Cuando como muchos bocadillos me salto las comidas regulares.
4. Hay periodos que parece que estuviera comiendo todo el tiempo, sin ninguna comida planeada.

### GRUPO 14

1. No pienso mucho acerca de tratar de controlar comer cosas que en realidad no deseo.
2. ALGUNAS VECES pienso acerca de tratar de controlar mi urgencia por comer.
3. MUCHAS VECES paso mucho tiempo pensando acerca de cuanto comí o acerca de tratar de no comer más.
4. SIEMPRE O CASI SIEMPRE estoy pensando en «comer o no comer». Siento que vivo para comer.

### GRUPO 15

1. No pienso que la comida sea «lo más importante».
2. Tengo antojos fuertes de comida, pero sólo por periodos cortos de tiempo.
3. HAY DÍAS que parece que no puedo pensar en otra cosa que no sea la comida.
4. LA MAYORÍA DE LOS DÍAS estoy preocupado(a) acerca de la comida. Siento como si viviera para comer.

### GRUPO 16

1. SIEMPRE O CASI SIEMPRE puedo distinguir si estoy físicamente hambriento(a) o no. Como lo suficiente como para satisfacerme.
2. A VECES me siento inseguro(a) de saber si estoy físicamente hambriento o no. Cuando pasa esto me es difícil saber qué tanto debo comer para satisfacerme.
3. Aunque pienso que debería saber cuántas calorías debo comer, no tengo ni la menor idea cual es la cantidad «normal» de comida para mí.

## Abbreviations

BE: binge eating
BES: Binge Eating Scale
BIS-15S: the Barratt Impulsiveness Scale-15S
BMI: body mass index
CFAs: confirmatory factor analyses
CFI: comparative fit index
EDI-3: Eating Disorder Inventory-3
FIML: full information maximum likelihood
mYFAS: Modified Yale Food Addiction Scale
RMSEA: root mean squared error of approximation
WLSMV: weighted least squares means and variances
YFAS: Yale Food Addiction Scale
